# Ruptured Superficial Femoral Artery Anastomotic Pseudoaneurysm after 30 Years

**DOI:** 10.1155/2019/1679214

**Published:** 2019-07-22

**Authors:** D. Baldwin, H. Mashbari, K. L. Chow, M. Sarhan

**Affiliations:** ^1^Department of General Surgery, University of Illinois at Chicago, Chicago, IL, USA; ^2^Department of General Surgery, Jazan University, Saudi Arabia; ^3^Department of Surgery, Division of Vascular Surgery, Advocate Christ Medical Center, Oak Lawn, IL, USA

## Abstract

**Introduction:**

Anastomotic pseudoaneurysms are a complication of vascular reconstructive surgery with the majority in the femoral region. Although rare, ruptured femoral anastomotic pseudoaneurysms have high mortality and require emergency surgery.

**Case Presentation:**

A 60-year-old male with a history of a left leg crush injury was treated with a superficial femoral artery interposition vein graft 30 years ago. He presented nowadays with a three-day history of severe pain in his left thigh. CT angiography demonstrated a ruptured anastomotic pseudoaneurysm with contrast extravasation into an intramuscular hematoma. He had significant scarring from his previous surgeries which made the leg hostile for an open repair. Therefore, percutaneous access selectively cannulated the left iliofemoral vasculature. An angiogram showed a distal superficial femoral artery pseudoaneurysm. Subsequently, two 10mmx15cm Viabahn covered stents (Gore & Associates, Flagstaff, AZ) were placed bridging healthy superficial femoral artery. A completion angiogram demonstrated no extravasation into the pseudoaneurysm. The patient recovered and was discharged home two days postoperatively.

**Conclusion:**

Ruptured femoral anastomotic pseudoaneurysms are traditionally repaired with open pseudoaneurysm excision and arterial reconstruction, although endovascular repair has been reported. Furthermore, most femoral anastomotic pseudoaneurysms form less than 10 years after initial operation. We present a unique case of ruptured superficial femoral artery pseudoaneurysm, 30 years after the initial operation. Endovascular stents offer effective treatment for ruptured anastomotic pseudoaneurysms.

## 1. Introduction

Pseudoaneurysms are localized arterial disruptions that communicate with an artery through a defect in the arterial wall. They are different from true aneurysms as they do not contain all three layers of the vessel wall. Pseudoaneurysms are commonly associated with penetrating trauma, iatrogenic injury during cardiac, endovascular, or radiological intervention, or arterial-graft anastomotic disruption. Other less common causes of pseudoaneurysm formation include bacterial, mycobacterial, or amoebic infection, inflammatory processes such as pancreatitis, or arterial degeneration from Marfans syndrome or fibromuscular dysplasia.

Complications from pseudoaneurysms include thrombosis, distal embolization, and potential free rupture. Rupture of anastomotic pseudoaneurysms results in high morbidity and mortality ranges from 20 to 46.6% [1, 2]. Patients need emergency surgery to prevent loss of life or limb. Traditional treatment involves open surgery with pseudoaneurysm excision and arterial reconstruction. However, the emergence of endovascular therapy has permitted another option for interventionalists in treating pseudoaneurysms. Endovascular techniques are limited with regard to availability and access but are invaluable in scarred or difficult to reach locations. To illustrate this, we present a unique case of ruptured superficial femoral artery pseudoaneurysm presenting 30 years after a reverse saphenous vein interposition graft repair in a hostile surgical field. The 30-year timeframe in which the femoral pseudoaneurysm ruptured and the inability to perform an open repair offered unique challenges.

## 2. Case Presentation

A 60-year-old obese male with a history of smoking, hypertension, diabetes, and chronic kidney disease had a previous left leg crush injury requiring a superficial femoral artery reverse saphenous vein interposition graft repair 30 years ago. Postoperatively, he suffered from chronic lymphedema and developed a left superficial femoral artery anastomotic pseudoaneurysm which was followed with serial imaging. Two months prior to presentation, ultrasound imaging showed the pseudoaneurysm had grown significantly to 6.8 x 5.7 x 5.0 cm, yet he refused surgery.

**Figure 1:**
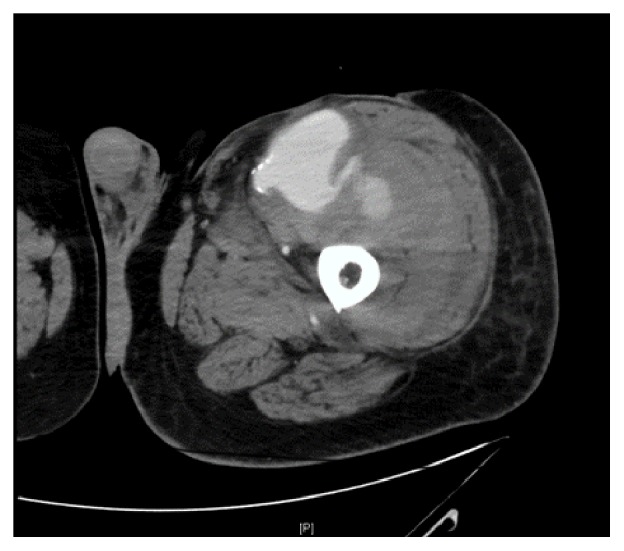
CTA of left lower extremity, axial images showing the anastomosis pseudoaneurysm with active extravasation.

**Figure 2:**
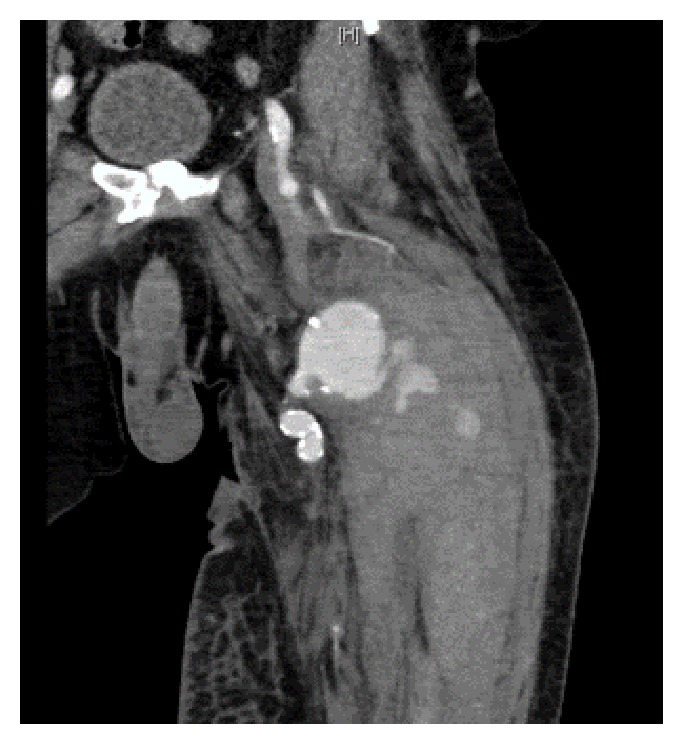
CTA of left lower extremity, coronal images showing the anastomosis pseudoaneurysm with active extravasation.

**Figure 3:**
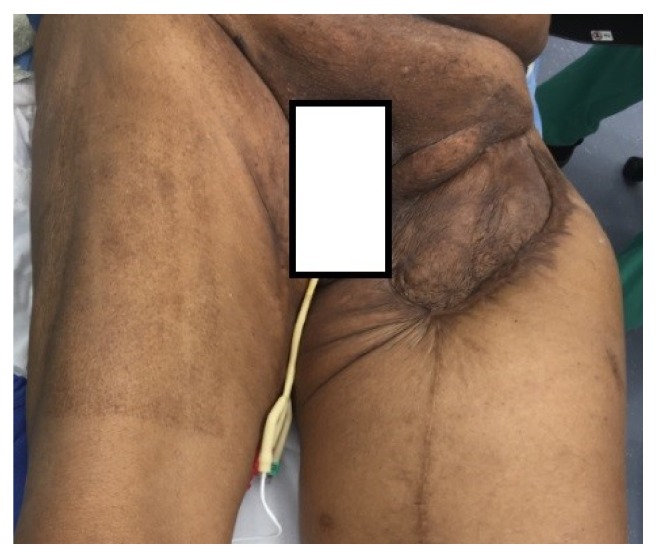
Left thigh of the patient on physical exam.

Upon presentation, the patient endorsed a three-day history of progressive, severe pain and swelling in his left thigh causing him to present to the emergency room. He presented with mild tachycardia, but other vital signs were within normal limits. CT angiography demonstrated a ruptured anastomotic pseudoaneurysm with contrast extravasation into an intramuscular hematoma measuring 12.4 x10.6 x 28.2 cm (Figures 1 and 2). On examination, he had significant scarring of the left groin from his previous surgeries and skin grafting which precluded open vascular repair (Figure 3). As such, we elected to perform an endovascular repair to avoid the hostile surgical field.

**Figure 4:**
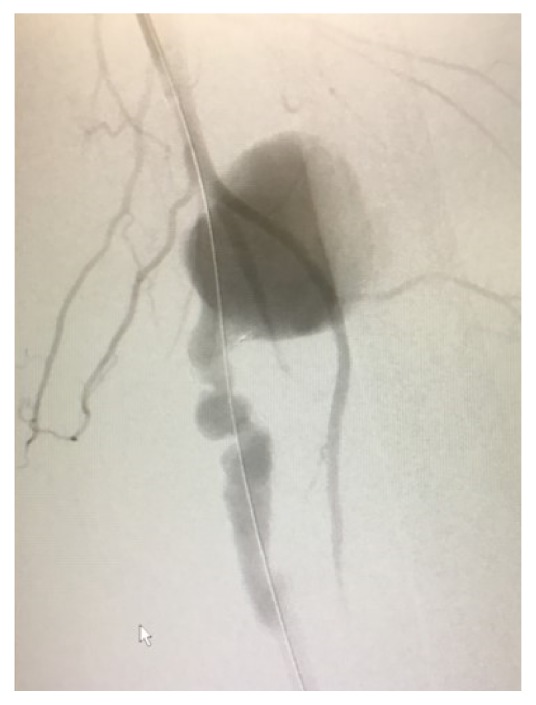
Intraoperative angiogram showing the left superficial femoral artery pseudoaneurysm with wire in place.

**Figure 5:**
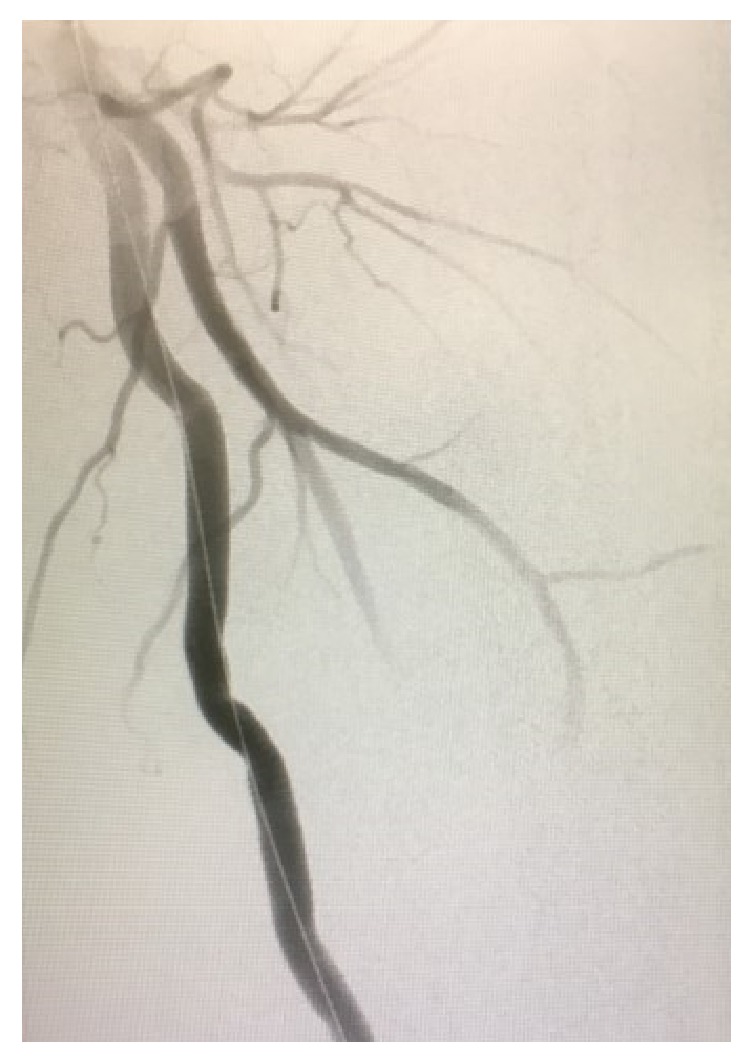
Intraoperative angiogram showing complete resolution the left superficial femoral artery pseudoaneurysm after stents placement.

Ultrasound guided percutaneous access was obtained via the right common femoral artery. A catheter was inserted and the left iliofemoral system was selectively cannulated using a guidewire and omniflush. A left lower extremity angiogram was obtained showing a pseudoaneurysm coming from the distal superficial femoral artery (Figure 4). At this point, a stiff wire along with an 11 French sheath was positioned at the normal superficial femoral artery just proximal to the pseudoaneurysm. Then two 10mm x 15cm Viabahn covered stents (Gore & Associates, Flagstaff, AZ) were placed with 8 cm of stent overlap bridging healthy superficial femoral artery proximally and distally. The stented segment was around 20 cm long. After 10mm balloon dilation of the stents, a completion angiogram demonstrated no extravasation to the pseudoaneurysm (Figure 5). We then made a small counter incision in the left groin to allow hematoma evacuation. The hematoma was not sent for culture. The patient had an uneventful recovery and was discharged home two days postoperatively. He required no postoperative anticoagulation. Although the patient had minimal follow-up, to our knowledge the stents remain patent.

## 3. Discussion

Current studies theorize anastomotic pseudoaneurysms form due to turbulent blood flow which progressively weakens the arterial wall promoting the development of anastomotic leaks [3]. The most common location for anastomotic pseudoaneurysms is the common femoral artery to bypass graft junction, which occurs in 0.8–2.2% of revascularization procedures [3]. In evaluating pseudoaneurysms, cultures of the graft and vessel wall are essential to exclude infection as an etiologic factor in the development of the anastomotic aneurysm [4]. Patients with known pseudoaneurysms are followed with serial ultrasounds and should be repaired if symptomatic.

Treatment of pseudoaneurysms depends on size, location, and feasibility. Arterial catheterization pseudoaneurysms less than 2cm can be observed, with most spontaneous resolving [5]. For larger or symptomatic pseudoaneurysms, nonoperative techniques include ultrasound-guided compression, thrombin injection, and coil embolization. However, these techniques are rarely used for anastomotic pseudoaneurysms. These are best treated with open surgery or endovascular repair [4, 5].

Skourtis et al. reported a case series of 49 patients with anastomotic pseudoaneurysms [2]. Most (85.7%) occurred at the femoral anastomosis site and emergency surgery for ruptured pseudoaneurysms was associated with 46.6% mortality [2]. In their case series, all patients were operated on with open surgery. Due to high mortality, free rupture of femoral anastomotic pseudoaneurysms is the most dreaded complication. Several papers have described ruptured femoral pseudoaneurysms mainly from infectious etiologies [1, 6, 7]. Klonaris et al. reported 6 patients with ruptured femoral artery anastomotic pseudoaneurysms secondary to infection. All patients were treated with emergency percutaneous covered stent deployment with surgical debridement in the following days to remove the infected pseudoaneurysm [1]. With the stent grafts covering the neck of the pseudoaneurysm, proximal and distal control were not needed, which limited the amount of open dissection. Patients were then placed on 6 weeks of intravenous antibiotics. At median follow-up of 14 months, there had been no indication of recurrent infection or pseudoaneurysm [1]. Until recently, open surgical repair had been the sole management option of such entities, despite its well-known limitations including scar tissue, distorted anatomy, and difficulty obtaining vascular control. This study highlights the expanding possibilities for endovascular covered stents.

Along with covered stents, there are several options for endovascular repair. Embolism with coils or liquid embolic agents, gelfoam, or vascular plugs have all been successfully used [8]. These options work well for most pseudoaneurysms, even in infected fields. Moulakakis et al. conducted a systematic review looking at infected pseudoaneurysms of the iliofemoral region treated with covered stent grafts. The most commonly used covered stents were Viabahn (22.9 %), Jostent (17.1 %), Fluency (14.3 %), and Wallgraft (14.3 %). Even in the infected field, stent patency was 94.3% at 16 months' follow-up with stent infection rate only at 3.4% [9].

In our patient, there was no evidence of infection leading to the pseudoaneurysm rupture. The most likely etiology was degeneration of the reverse saphenous vein bypass conduit. The incidence of femoral anastomotic aneurysms occurs less frequently with saphenous vein grafts than synthetic vascular grafts, a reflection of more complete healing of autogenous tissue [4]. Marković et al. examined 87 patients with pseudoaneurysms following bypass surgery. The causes were primarily arterial degeneration (66%) and infection (25%). These patients presented mainly with ruptured pseudoaneurysm, thrombosis, chronic limb ischemia, or acute limb ischemia. Most patients presented between 2 and 5 years after the initial operation and were treated with open repair. Furthermore, only 12.6% of patients presented after 10 years [10].

The timeframe for development of anastomotic pseudoaneurysms varies based on study but is reported between 5 and 9.2 years after primary operation [7, 10]. Skourtis et al. in their case series of 49 patients with anastomotic pseudoaneurysms reported that mean time of pseudoaneurysm developed to be 6.8 years [2]. In a larger study by Levi et al. with 76 patients who had femoral anastomotic pseudoaneurysms, the interval between the primary operation and the operation for the femoral pseudoaneurysm was 9.2 years, with a range from 1 month to 26 years [7]. To our knowledge, we present the first noninfected anastomotic femoral pseudoaneurysm rupture after 30 years.

Such late presentations of anastomotic pseudoaneurysms from venous conduits are rare. It is unclear if this is due to durable anastomotic integrity or due to the unfortunate reality that many vascular patients do not survive for 30 years with their bypass. The etiology of long-term failure is still unclear but a combination of progression of the host's underlying disease, increased graft intimal hyperplasia, and the development of atherosclerosis-like lesions within the vein graft appears to be the principal cause [11]. However, as evidenced by this case report, there may be an upper limit to anastomotic integrity even amongst well established and long-standing anastomoses. This case report may serve clinicians as evidence for vascular anastomosis surveillance even up to 30 years.

## 4. Conclusion

Ruptured femoral anastomotic pseudoaneurysms are rare but have high mortality. Traditional repair involves open pseudoaneurysm excision and arterial reconstruction, although endovascular repair has been reported. Furthermore, most femoral anastomotic pseudoaneurysms form between 5 and 9.2 years after initial operation. To our knowledge, we present the first noninfected anastomotic femoral pseudoaneurysm rupture after 30 years successfully treated with covered endovascular stents. The late presentation of rupture adds to the literature and may serve clinicians as evidence for vascular anastomosis surveillance even up to 30 years.
